# Influence of barley straw (*Hordeum vulgare* L.) extract on phytoplankton dominated by *Scenedesmus* species in laboratory conditions: the importance of the extraction duration

**DOI:** 10.1007/s10811-012-9900-7

**Published:** 2012-09-20

**Authors:** Wojciech Pęczuła

**Affiliations:** Department of Hydrobiology, University of Life Sciences, ul. Dobrzańskiego 37, 20-062 Lublin, Poland

**Keywords:** Barley straw, Extract, Phytoplankton, Scenedesmus

## Abstract

The response of a natural phytoplankton assemblage dominated by algae of the genus *Scenedesmus* to the addition of barley straw extract was studied in a laboratory experiment. The aim of the study was to compare the inhibiting effect of water extracts obtained by soaking the straw for 1, 2 and 3 months. We analysed the response of four species, *Scenedesmus subspicatus*, *Scenedesmus ecornis*, *Scenedesmus quadricauda* and *Scenedesmus acuminatus*, during 14 days of their exposure to different types of barley straw extract. *S. subspicatus* and *S. ecornis* responded with decreasing numbers only to the addition of the 3-month solution (ANOVA; *F* = 290.1, *p* <0.001; and *F* = 11.8, *p* <0.01, respectively); the two other species were inhibited by all types of extracts. The results indicate the need for more research on the importance of extraction duration to the inhibitory abilities of barley straw which can be applied in the management of water quality in water bodies.

## Introduction

Although the mass occurrence of planktonic algae in lakes and reservoirs has been for many years one of the main problems in water quality management, there is no generally accepted, inexpensive and environmentally safe method of reducing this phenomenon. One promising technique is an application of barley straw, which has been used in Britain since the 1980s (Welch et al. 1990). The idea of a cheap, simple and safe method of reducing algal blooms caused extensive research on its application (for review, see Ó hUallacháin and Fenton 2010). There have been attempts to apply barley straw directly in channels (Welch et al. 1990; Caffrey and Monahan 1999), ponds (Boylan and Morris 2003; Butler et al. 2005), lakes (Harriman et al. 1997; Wiśniewski 2002) and drinking water reservoirs (Everall and Lees 1997; Barett et al. 1999). Laboratory research confirmed the inhibitory effects of barley straw on selected species of algae (Gibson et al. 1990; Newman and Barrett 1993; Martin and Ridge 1999). After several years of research, it is recognised that the method has limitations and drawbacks. The results of some work shows that the use of barley straw in some cases has no effect on selected species of phytoplankton (Boylan and Morris 2003; Brownlee et al. 2003; Ferrier et al. 2005) or can even stimulate the algae, for example, the bloom-forming cyanobacteria *Planktothrix aghardii* and *Microcystis aeruginosa* (Molversmyr 2002). Another problem is the dose of straw that should be used in a water body. The effective dose that limits algal blooms is estimated at 250–300 kg of straw ha^−1^ (McComas 2003), which in turn makes this method neither cheap nor easy to use in large lakes or reservoirs. Therefore, some laboratory studies have focused on the use of aqueous extract of barley straw, which would facilitate its use in ponds with a smaller area. So far, the main approaches in the research seem to be the impact of the extract on various species of algae (Ball et al. 2001; Brownlee et al. 2003; Terlizzi et al. 2002; Ferrier et al. 2005) or the chemical composition of the extract (Ridge and Pillinger 1996; Waybright et al. 2009; Murray et al. 2010). An important practical issue required for the proper use of straw is the time needed to obtain an inhibitory effect (Ó hUallacháin and Fenton 2010). Observations on the barley straw application in field studies have shown that the washout time of inhibiting substances is usually several months (Welch et al.1990; Everall and Lees 1997; Harriman et al. 1997.). However, the problem of time needed to achieve an effect on algae is rarely considered in laboratory studies. Gibson et al. (1990) studied the effect of the straw decomposition degree on its suppressing ability, stating that 6-month incubation is most effective on filamentous algae. There are no similar studies concerning barley straw extracts and their effects on phytoplankton, especially those taking into consideration short-time extraction. The aim of this research was to compare the anti-algal activity of the extract obtained by soaking the barley straw for varying lengths of time (1–3 months) and to test the hypothesis that a short-time extraction can also produce a liquid with algae-suppressing ability. The effect of the extract was tested on a natural phytoplankton assemblage composed mainly of green algae of the genus *Scenedesmus* in laboratory experiment conditions.

## Materials and methods

For the extraction, we have used barley straw (*Hordeum vulgare* L.) obtained from a 2009 crop and kept under dry conditions for a period of 10 months after harvesting. We prepared three sets of aquaria consisting of three tanks, each with a capacity of 16 L. The same quantity of 80 g of straw was placed in a set of three at monthly intervals, so the resulting dose of straw amounted to 5 g of straw L^−1^ in each aquarium. The tanks were filled with dechlorinated tap water and then continuously aerated using a standard aquarium aerator and kept at room temperature (~20–22 °C) for 1, 2 or 3 months. After the extraction ended, 0.2 L of each solution was filtered through a cellulose paper filter (basis weight of 84 g m^−2^) followed by a single boiling. This process yielded three types of solutions after extraction times of 1, 2 and 3 months, marked as 1m, 2m and 3m, each with three replicates.

For the experiment, unfiltered water from a small eutrophic pond was collected in late summer. The phytoplankton consisted mainly of green algae with various species of the dominant genus *Scenedesmus*. Water with seston was placed in an aquarium and kept at room temperature, aerated and illuminated with a fluorescent lighting with a colour temperature of 6,500 K (daylight) and giving an irradiance of 25 μmol photons m^−2^ s^−1^ (measured by Li-Cor LI 250A Light Meter) 12 h day^−1^ for 14 days. The culture was supplied with an initial dose of phosphorus and nitrogen (0.68 mg P L^−1^ and 8.43 mg N L^−1^).

The influence of the extract on the phytoplankton assemblage was tested in 24 PET bottles with a capacity of 1.5 L. The same amount of algal culture (0.4 L) was poured into 18 bottles, as well as 0.2 L of different types of extracts in duplicate. This way, each type of extract (1m, 2m and 3m) was tested in a total of six experimental replicates. The dose of the extract was calculated to correspond to 1,660 g of barley straw added to 1 L of water. Six bottles served as controls and were filled with 0.4 L of enriched seston and 0.2 L of dechlorinated water. The experiment was conducted in September 2010 and lasted 14 days. Bottles were kept under the same conditions as the previous aquaria of algae and were shaken every day. Temperatures during the experiment ranged from 20.7 to 21.6 °C.

Initially (in the control suspension) and at the end (in all bottles) of the experiment, pH and conductivity using the YSI 556 Multi Probe (MPS, USA) and total organic carbon concentration using the UV analyser (Pastel, France) were measured. Species composition of phytoplankton in 100-mL samples taken at the beginning and at the end of the experiment was based on microscopic analysis using an inverted microscope and the Ütermohl method (Vollenweider 1969). Abundances are expressed as colonies (coenobia) mL^−1^. In order to determine the significance of differences between the control and experimental assemblages, ANOVA analysis with Tukey's test was performed using Statistica 6.0 software (Statsoft Inc., USA).

## Results

The phytoplankton community after 14 days in the laboratory consisted mainly of green algae from the genus *Scendesmus* as well as small numbers of *Coelastrum* and *Ankistrodemsus* species. The genus *Scendesmus* was represented by four species: *Scendesmus subspicatus* Chodat (dominant species, 45–50 % of total phytoplankton abundance), *Scendesmus acuminatus* (Lagerheim) Chodat, *Scenedesmus quadricauda* (Turpin) Brébisson and *Scendesmus ecornis* (Ehrenberg) Chodat. These four species were included in the subsequent analyses and the others were omitted because their abundances were too low (<1,000 cells or colonies mL^−1^).

**Fig. 1:**
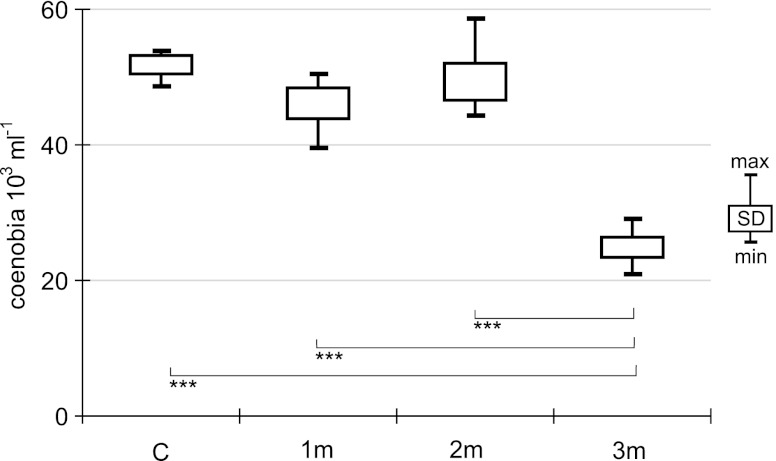
Abundance of *S. subspicatus* in experimental and control bottles after 21-day exposure to various barley straw extracts (*N* = 6; *C* control; *1m*, *2m*, *3m* extracts after 1, 2 or 3 months of extraction; *triple asterisks p* < 0.001; one-way ANOVA)

The initial number of *S. subspicatus* was 52.5 ± 2.9 × 10^3^ coenobia mL^−1^. After 21 days, numbers of this species in control 1m and 2m tanks were on similar level: 51.8 ± 2.7 × 10^3^ colonies mL^−1^ (control), 46.1 ± 4.4 × 10^3^ colonies mL^−1^ (1m) and 49.4 ± 5.5 × 10^3^ colonies mL^−1^ (2m) (Fig. 1) and statistically did not differ one from the other (ANOVA; c-1m, *F* = 7.348, *p* > 0.01; c-2m, *F* = 0.357, *p* > 0.05; 1m–2m, *F* = 2.129, *p* > 0.05). In the tank receiving the 3-month extract (3m), abundances were lower at 24.8 ± 3.0 × 10^3^ coenobia mL^−1^. Analysis of variance showed that only the 3m tank differed significantly from the control and other extract treatments (ANOVA, *F* = 290.1, *p* <0.001 for control; *F* = 95.3, *p* < 0.001 for 1m; and *F* = 103.0, *p* <0.001 for 2m).

**Fig. 2:**
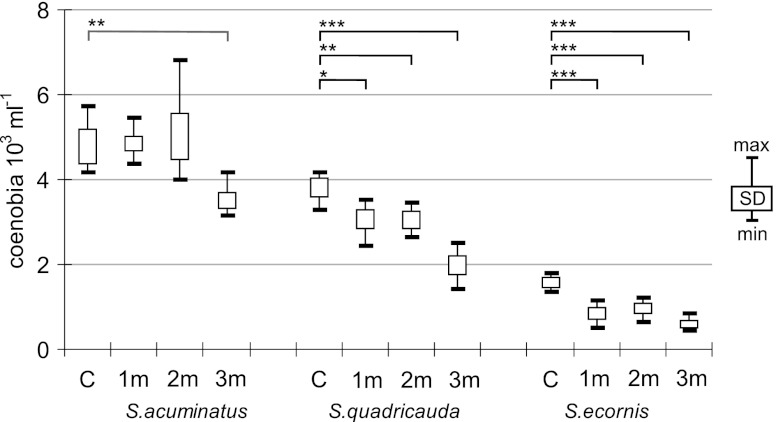
Abundance of *S. acuminatus*, *S. quadricauda* and *S. ecornis* in experimental and control aquaria (*N* = 6; *C* control; *1m*, *2m*, *3m* extracts after 1, 2 or 3 months of extraction; single asterisk *p* < 0.05; double asterisks *p* < 0.01; triple asterisks *p* < 0.001; one-way ANOVA)

The other species of *Scenedesmus* were less abundant at the beginning of the experiment: *S. acuminatus*, 4.2 ± 1.0 × 10^3^ coenobia mL^−1^; *S. quadricauda*, 4.0 ± 1.2 × 10^3^ coenobia mL^−1^; and *S. ecornis*, 1.4 ± 0.4 × 10^3^ coenobia mL^−1^. The exposure to the extracts caused species-specific effects (Fig. 2). *S. acuminatus* abundances were reduced only in the tanks with 3-month extract, as noted above for *S. subspicatus*, and the difference between the numbers in the controls and 3m tanks was statistically significant (*F* = 11.8, *p* <0.01). The numbers of *S. quadricauda* were statistically lower in 2m and 3m bottles (*F* = 10.3 and *F* = 50.8, respectively; *p* <0.01), although the population reduction was also observed in 1m bottles (*F* = 8.5, *p* = 0.015). *S. ecornis* had reduced numbers after exposure to all types of extracts (*F* = 28.5 for 1m, *F* = 22.6 for 2m and *F* = 70.6 for 3m; *p* <0.01).

Straw extract added to the bottles induced changes in conductivity, pH and concentration of dissolved organic carbon (Table 1). The older the extract added to the bottles, the higher the conductivity of water, and the difference between the controls and 3m extract after exposure was more than 100 μS cm^−1^. The pH values, which were relatively high in the control bottles (pH 8.6–8.9) were reduced after adding the extract and at the end of the experiment were within the range of pH 8.4–8.5 (mean values). However, the organic carbon content in the bottles with added extract was nearly twice the control concentrations and ranged from 21.8–22.7 mg L^−1^ (mean values).

**Table 1:** Mean (±standard deviation) values of conductivity, pH and concentration of total organic carbon in control samples and samples with 1-, 2- and 3- months extracts

	C	1m	2m	3m
EC (μS cm^−1^)	529.6 ± 5.9	592.2 ± 26.6	638.8 ± 24.3	646.5 ± 22.1
pH	8.8 ± 0.1	8.4 ± 0.3	8.5 ± 0.2	8.5 ± 0.2
TOC (mg L^−1^)	13.2 ± 0.7	21.8 ± 1.0	22.2 ± 3.2	22.7 ± 3.7

*TOC* total organic carbon; *C* control; *EC* conductivity; *1m*, *2m*, *3m* extracts after 1, 2 or 3 months of extraction

## Discussion

The exposure of the tested species to different types of extract caused either no response or a reduction in population numbers. The 3-month extract caused a reduction in the number of all four *Scendesmus* species, while in the case of 1-month and 2-month extracts, the response of algae was also observed in *S. quadricauda*, *S. ecornis* and *S. acuminatus*.

*S. subspicatus* proved to be resistant to 1m and 2m extracts, but its reaction to the 3-month extract was most evident among other species of the genus. Interestingly, other studies show that this species may be stimulated by decomposing barley straw (Martin and Ridge 1999). The authors applied wet barley straw (3–6 months of age) directly in doses several times higher than in our study (4,000 g of straw m^−3^), but the exposure lasted only 4 days, which corresponds to the addition of small quantity of extract. Murray et al. (2010) studied the effect of phenolic compounds, which are usually released from decomposing barley straw on three species of algae, including two chlorophytes. Although algistatic effects were observed in some of these compounds, *S. subspicatus* appeared the most resistant to any inhibition. There are also some reports on *S. quadricauda* responses to barley straw addition. Ferrier et al. (2005) have ranked this species among algae resistant to barley straw extract; their experiment showed a slight increase in cell numbers under 2-week exposure to 2-month-old extract. These reports may not be in conflict with the results obtained in our experiment if we put forward a hypothesis that *S. subspicatus* and *S. quadricauda* respond only to long-term exposure of rotting straw. A similar view is presented by Choe and Jung (2002), who found that certain species, although inhibition-responsive at high doses of plant extracts, may show an increase on exposure to lower extract concentrations. Our results also confirm the thesis that the inhibitory effect of straw on algae is not universal over all taxa but rather depends on particular species (Brownlee et al. 2003; Ferrier et al. 2005).

We are aware that experiments performed on a poorly controlled phytoplankton assemblage derived from a natural habitat, instead on pure algal cultures, may give results whose interpretation is limited. In such a community, the growth and development of individual species is affected by many factors, including trophic interactions with other organisms. For example, the growth of bacteria caused by an increase in dissolved organic matter derived from straw may result in reduced availability of nutrients to algae as a result of competition (Klug 2005). We did not monitor the nutrient concentrations nor dynamics during the experiment, which could be important in the reduction of algae growth, nor did we examine the chemical composition of the extract that inhibits the algae. The experimental results showed only that the extract addition changed some of the basic water chemistry, such as conductivity (increase), pH (decrease) and, most obviously, the content of total organic carbon (increase). However, the aim of the research was to determine the importance of the straw extraction duration for inhibiting algae; hence, the main conclusions can only be applied to this goal.

Why is the extraction time important? It is generally accepted that algal growth inhibition by barley straw is associated with the secretion of various chemical substances as algaecides, among them phenolic substances that play a key role (Pillinger et al. 1994; Waybright et al. 2009). These substances are derived from microbial degradation of the lignin in the straw tissues, performed mainly by fungi (Pillinger et al. 1992). The most active substances begin to be formed only after 3 months of straw incubation in water (Pillinger et al. 1994; Ferrier et al. 2005), which is probably due to the time needed by a fungal community to colonise the substrate (Murray et al. 2010). Moreover, initially, lignin is not decomposed as this process occurs only under conditions of nitrogen deficiency, which is rare in eutrophic waters (Kirk and Farrell 1987). At first, intensive decomposition of cellulose occurs in straw tissues; Murray et al. (2010) found a total loss of cellulose in barley straw after 5 months of incubation in water. Unfortunately, there are no studies that have attempted to identify the chemical compounds which are products of the early stage of barley straw rotting (including cellulose decomposition), although it is suspected that they are different from those produced in later phases (Ferrier et al. 2005).

As both the rotting process and release of compounds is connected with microbial activity, physical factors, like temperature, should also be considered. Previous research in which barley liquor was prepared in laboratory was very similar in terms of the temperature used. The temperature of extraction in experiments was stated to be between 18 and 25 °C or ‘room temperature’, but any attempts are known to measure the impact of this factor on the extract activity (Gibson et al. 1990; Martin and Ridge 1999; Ball et al. 2001; Terlizzi et al. 2002; Ferrier et al. 2005). Another question is the light availability during rotting of the straw. If the decomposition and release of the anti-algal compounds are only driven by fungi, the light conditions during the extraction are not so significant, so the majority of extracts in mentioned research were conducted in the dark. However, some researchers point that rotting straw can release precursive phenolic compounds which can undergo phototransformation in oxygenated conditions, which can result in production of hydrogen peroxide and other phytotoxic substances (Everall and Lees 1997). If we take into consideration, that the use of barley straw should be a technique applicable in lakes and reservoirs, laboratory studies on straw extraction should mimic field conditions (Ó hUallacháin and Fenton 2010). This implies that both temperatures and light conditions should be similar to those in freshwaters during vegetation season.

The results of the presented research showed that 3-month extraction of barley straw produces the best results in the inhibition of all species of *Scenedesmus*, but the inhibiting effect can be achieved in some species by extraction lasting 1 month. This confirms that the effect of duration of barley straw extraction on its inhibiting effect is an important problem and that research on this topic should be continued. They should include not only the effects of time but also the role of light, temperature and oxygen content, as all these factors can be fixed with microbial activity and other mechanisms responsible for leaching anti-algal compounds. Such knowledge can be transferred into practice in the water quality management in lakes and reservoirs, by the determination of not only the adequate doses but also the conditions in which the straw should be applicable.
